# Supplementary material for: Structural Basis of Activity of HER2-Targeting Construct Composed of DARPin G3 and Albumin-Binding Domains
Source: Int J Mol Sci. 2024 Oct 22;25(21):11370. doi: 10.3390/ijms252111370 (PMC11545695; doi:10.3390/ijms252111370)
Supplement: Supplementary file 1 [file ijms-25-11370-s001.zip › ijms-3244987-supplementary.pdf]

Supplementary materials for:

NMR study of mixture of free G3 and ABD

According to the ^{1}H-NMR spectra, the free G3 and ABD interact via non-covalent association in solution. Despite the observed differences, the distribution and broadening of 1H signals (Figure S1B,C) of the G3/ABD complex is somewhat closer to those in the spectrum of conjugated G3-ABD, while some signals (e.g., a low-field signal at 11.3 ppm of side chain hydroxyl group of Tyr of G3) are similar to ABD-G3. Notably, the signal splitting on the two components with similar occupancy ratio of the major and minor conformations is also observed (e.g., a low-field amide signal near 9.6 ppm), presumably due to the interactions of nonconjugated G3 and ABD via alternative interfaces. Thus, the interactions of free G3 and ABD are similar but with some differences for the two DARPin conjugates. After the addition of HSA (in equimolar proportion), the complex was destroyed and signals of free G3 appeared in the ^{1}H-NMR spectrum (Figure S1C).

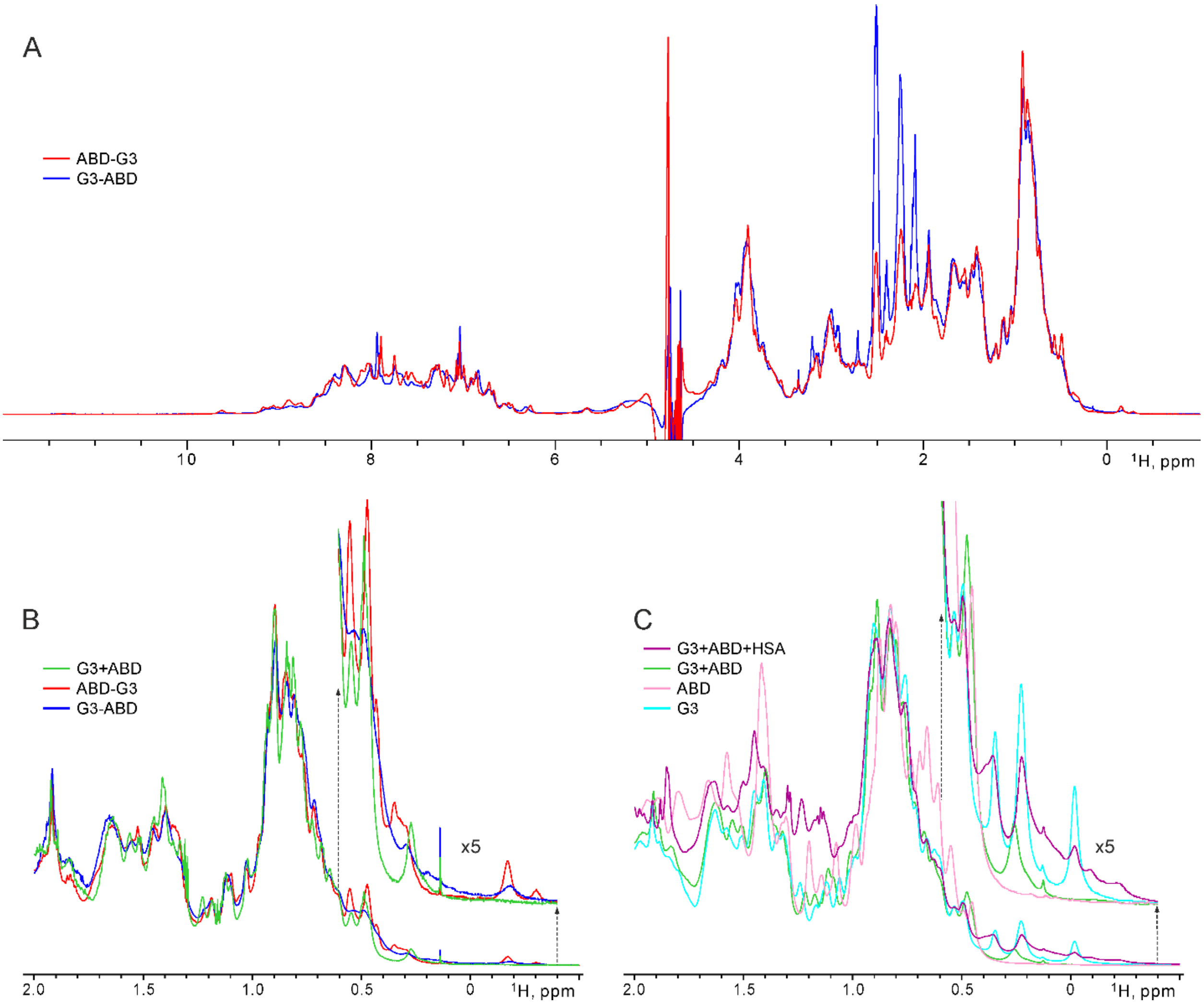

Figure S1. ^{1}H-NMR spectra of the DARPins conjugates and free G3.

(A) Overlaid ^{1}H-NMR spectra of the DARPins conjugates G3-ABD (in blue) and ABD-G3 (in red). **(B)** The characteristic regions with the methyl group signals of overlaid ^{1}H-NMR spectra of the DARPins G3-ABD (in blue), ABD-G3 (in red) and complex of nonconjugated G3 and ABD (in green). **(C)** The characteristic regions with the methyl group signals of overlaid ^{1}H-NMR spectra of free G3 (in cyan), free ABD (in pink), complex of nonconjugated G3 and ABD (in green) and after addition of HSA (in equimolar proportion) to the complex (in magenta). The high-field regions of the spectra are zoomed.

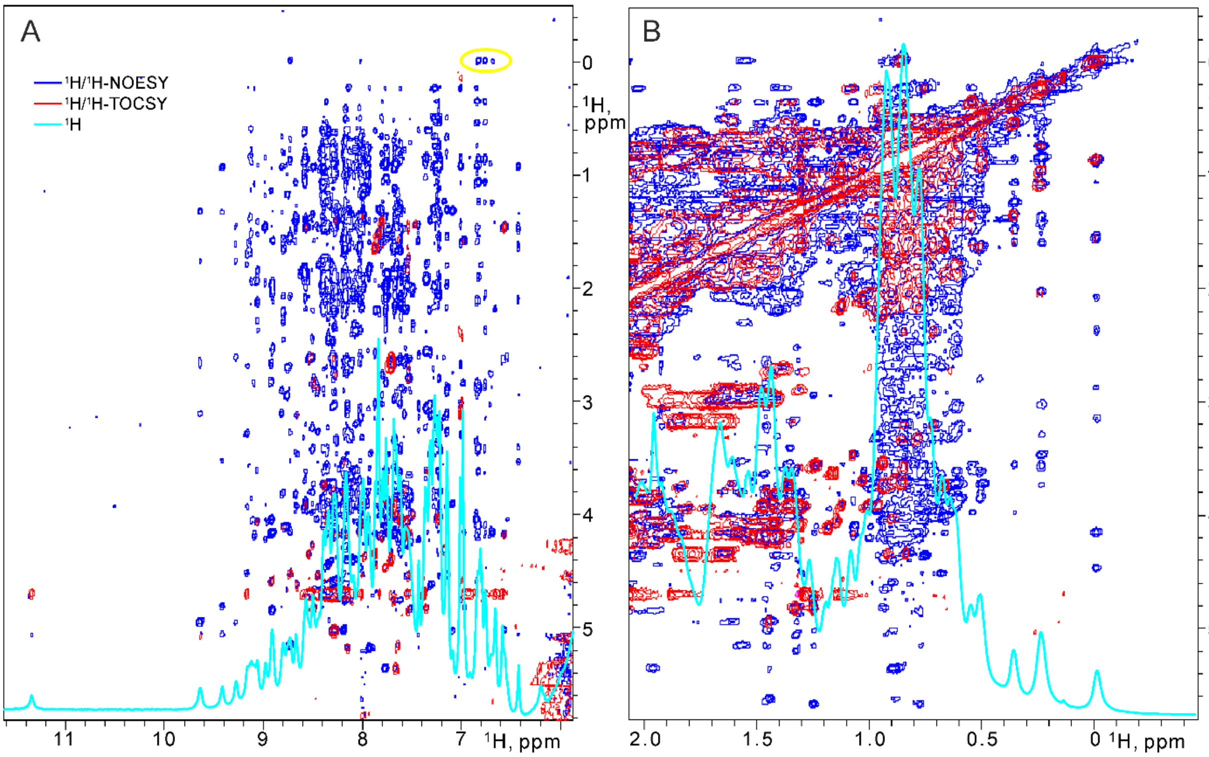

Figure S2. Two-dimensional ^{1}H-NMR spectra of free G3.

Overlaid ^{1}H/^{1}H-NOESY (in blue) and ^{1}H/^{1}H-TOCSY (in red) spectra acquired mixing times of 150 and 60 ms, respectively, are presented. The characteristic regions with the signals of amide and aromatic groups (**A**) and methyl groups (**B**) are zoomed. *In bottom*, the corresponding ^{1}H-NMR spectrum is shown in cyan. The spectra characterize the protein conformation even without a specific assignment of chemical shifts. For example, according to the NOE cross-peak pattern surrounded by a yellow oval (A), the methyl group of one of the Ile residues, which has a pronounced high-field ^{1}H-signal near 0 ppm (B) that undergoes an additional high-field shift to -0.2 ppm in the ^{1}H-NMR spectra of DARPins G3-ABD and ABD-G3 (Figure 2B), is spatially adjacent to the aromatic ring of Phe, which has ^{1}H-signals in the range of 6.8-7.0 ppm (A).

G3/ABD interactions: results of MD simulations

The structural features of the G3-ABD and ABD-G3 chimeras in an aqueous environment were investigated using two independent computational approaches in the all–atom representation - MD calculations in an explicit water and MC conformational search using the implicit solvent model developed by the authors earlier.

Hydrophobic interface of G3 with ABD (np-site).

In four 200-ns MD trajectories, where domains were spatially close by their HER2 (G3) and HSA-binding (ABD) motifs, the corresponding domains of both chimeras interact tightly all the time of MD calculation. Two out of three α-helices (C-terminal in G3-ABD molecule) of ABD domain are involved in interaction with DARPin. The degree of MHP complementarity (it is mainly hydrophobic) of the contacting molecular surfaces of the two interacting domains in both np-MD and experimental models of the complexes is more than 50%.

As shown in the Table S1, integral parameters of the "hydrophobic" binding interface (np-site) such as the contact area, hydrophobic and electrostatic atom–atom contacts, along with H-bonds and a number of stacking pairs are quite similar for both molecules. In the low-energy model of the G3-ABD complex obtained via MC search, the domains' interaction interface is similar to the "hydrophobic" one observed in MD simulations (see Figure 3B, G3- and ABD-residues highlighted in magenta). It should be noted that spontaneous formation of G3-ABD complex with the hydrophobic interface when starting from non-interacting domains was not observed in MD calculations. In contrast, such associates were obtained in the result of MC conformational search, which permits exhaustive sampling of the phase space. Being rather rough in comparison with the explicit model of aqueous solution used in MD, such approach additionally supports the possibility of the complex formation even without any initial guess about feasibility of this scenario.

```
149        160  .     170       180       190
|          |    .     |         |         |
LAEAKVLANRELDKYG-VSDFYKRLINKAKTVEGVEALKLHILAALP  ABD-domain
LAEAKVLANRELDKYG-VSDYYKNLINNAKTVEGVKALIDEILAALP  1gjs(res:20-65)
* :**  *  **.* * .**** : ******** *:***..**.*..  *-identical residues
LKNAKEDAIAELKKAGITSDFYFNAINKAKTVEEVNALKNEILKAHA  PDB ID:2vdb(res:9-53)
               *fhh ff  f  e*e* e   *f  ef e**    HSA-motif[1]
hhhhhhhhhhhhh      hhhhhhhhh   hhhhhhhhhhhhhhh    helical residues
```

Figure S3. Protein sequence alignment of ABD-module with its structural template (PDB ID: 1gjs) and albumin-binding domain (from *Finegoldia magna*) as part of the complex with albumin (PDB ID: 2vdb). [1]interface residues (from 2vdb) involved into the H-bonding ("*h*"), hydrophobic ("f") and electrostatic ("e") interactions with Albumin molecule.

Table S1. Structural parameters[a] of binding interfaces of G3 /ABD complexes.

	G3-ABD "np-site"	***ABD-G3 "np-site"***	***G3-ABD "p-site"***	***ABD-G3 "p-site"***
**Number of MD-runs**	**2**	**2**	**4**	**5**
**Starting orientation of the domains**	Zdock-complex	Zdock-complex	Distantly located domains(>30Å)	Distantly located domains(>30Å)
G3 - ABD				
**Contact area, Å^2**	581 ± 110	518 ± 74	240 ± 80	340 ± 97
**Atom-atom contacts[b]**	97 ± 18	84 ± 11	37 ± 15	48± 16
**Hydrophobic contacts**	32 ± 7	27 ± 5	10 ± 4.0	16 ± 6
**H-bonds[c]**	2.8 ± 1.3	1.6 ± 1.0	2.3 ± 1.2	1.7 ± 1.1
**Electrostatic contacts**	1.3 ± 0.8	0.1 ± 0.4	1.7 ± 1.2	0.7 ± 0.9
**Stacking interactions**	0.9 ± 0.7	0.9 ± 0.9	-	0.4 ± 0.5
**Mobility[d]: G3**	1.3 ± 0.3	1.2 ± 0.2	1.5 ± 0.3	1.3 ± 0.4
**Mobility: ABD**	1.3 ± 0.2	1.4 ± 0.3	1.4 ± 0.3	1.3 ± 0.3
**Population[e],%**	97	96	37/29	71/65
**HSA-motif[f]**	79 ± 11	69 ± 11	28 ± 5 / 14 ± 9	55 ± 9 / 37 ± 4
**Predicted ΔG[g]**	-5.5 ± 0.5	-4.5 ± 0.5	-3.6 ± 0.5 / -4.6 ± 0.5	-4.0 ± 0.3 / -4.3 ± 0.4
**Predicted ΔG_*L*[h].**	-6.6 ± 0.6	-5.7 ± 0.7	-4.7 ± 0.4 / -5.2 ± 0.7	-6.6 ± 0.7 / -5.9 ± 0.8
Linker–G3				
**Atom-atom contacts**	31 ± 9	42 ± 15	27 ± 12	47 ± 17
**H-bonds**	2.4 ± 1.5	3.8 ± 2.2	1.1 ± 1.1	3.3 ± 1.7
Linker–ABD				
**Atom-atom contacts**	26 ± 5	9 ± 5	20 ± 8	20 ± 14
**H-bonds**	2.4 ± 1.2	0.3 ± 0.6	2.5 ± 1.5	1.0 ± 1.3

[a]Data (average values ± standard deviations) were given by averaging over all MD states, where domains are in contact;

[b]Atom-atom contacts, electrostatic and hydrophobic contacts between heavy atoms of the corresponding protein domains with a cut-off distance 6 Å (4 Å for hydrophobic contacts) in terms of a number of different residue-residue pairs;

[c] Number of Hydrogen bonds between protein domains;.

[d] Root-mean-square deviation (RMSD) values (in Å) of the backbone atoms from the starting conformations;

[e] Population of MD-states for which ΔG values and the degree of involvement of the HSA-motif of ABD in G3 binding are estimated. For complexes with p-sites, two most populated p-sites for each chimera are presented (populations are given for corresponding trajectories and separated by a slash);

[f]Surface area (% from the total surface area of HSA-binding motif (PDB:2vdb)) of HSA-residues that in contact with G3;

[g] Binding affinity ΔG (in *kcal/mol*) is calculated using the web-server PRODIGY (see Section Methods for details);;
[h] ΔG calculated taking into account the interaction of the Linker with G3 domain.

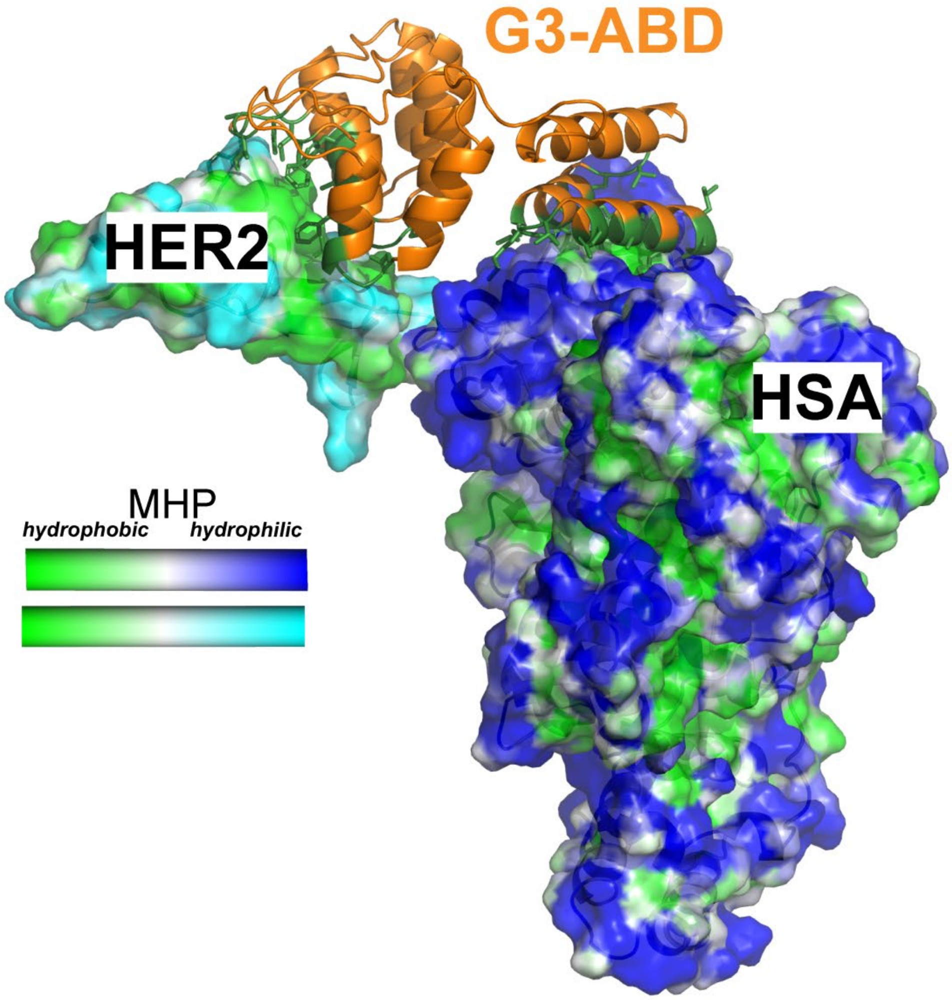

Figure S4. MD simulations: molecular reconstruction of HER2/G3-ABD/HSA complex. HSA can bind G3-ABD without dissociation of its modules (G3 and ABD). At the same time, bound HSA may sterically interfere with the binding of the G3-ABD complex to the HER2 receptor site. The molecular reconstruction is made based on the superposition of G3 and ABD-modules taken from the experimental models (PDB ID:1hrn, 2vdb) with the corresponding model of G3-ABD domains obtained in MD simulations in water. The molecular surfaces of both experimentally-derived models of the HER2 (subdomain IV, PDB:1hrn) and HSA (2vdb) are colored according to the values of the molecular hydrophobicity potential (MHP). The corresponding scales are given with the spectral bands. The HER2/np- and HSA- interface residues of G3 and ABD domains within the G3-ABD chimera (snapshot from w-MD trajectory) are given in stick representation and colored in green.
